# Supplementary material for: Group B Streptococcus CRISPR1 Typing of Maternal, Fetal, and Neonatal Infectious Disease Isolates Highlights the Importance of CC1 in In Utero Fetal Death
Source: Microbiol Spectr. 2023 Jun 21;11(4):e05221-22. doi: 10.1128/spectrum.05221-22 (PMC10434043; doi:10.1128/spectrum.05221-22)
Supplement: Supplemental file 1 — Table S1. Download spectrum.05221-22-s0001.docx, DOCX file, 0.05 MB [file spectrum.05221-22-s0001.docx]

Terminal direct repeat and main ancestral spacers according to major clonal complexes. Nucleotide sequence according to the graphical representation for each spacer and for each DRT is presented below the table.

| **Clonal complex** | **Spacers** | **DRT** |
| --- | --- | --- |
| CC17 |  |  |
| CC10 |  |  |
| CC23 |  |  |
| CC1 |  |  |
| CC19 |  |  |
| CC22 |  |  |
| CC388 |  |  |

Nucleotide sequence according to the graphical representation for each spacer:

 TATTTGATAGCGGTAACGGGTCATATACAA TGGTGGTATTTATAATGTACGAGCAAATCG

 GATAAAAAGTGGGAGCTGAATTAAAAGGCA ATTTGAACGATTTTTATATTCCTGATATGT

 TTCTATCTTCTGAAGATATTTCACAAGTGA TCAGCGAGATGCTCTAAGTAAGCATGTTGA

 TCTTCTTTTTAATTCTTCTAACACTCCATC ATCTTCTTTTGACCTAACAAAAGGATATGT

 AGAGGGGAAAATATCAATGCCGAATGCTGA GATGGTACAAAATCATTTGTTGGTACTGAT

 TTTTTACCAATGCTTCCATATCGCTTATAT TACTTGACGAATTGAAGATGACGGAATTTA

 TGGTTATACATTTACTAATCCATCAGCATT AAGCTAATTCTCATCTCACCGAGATGGATA

 TTTTACCAATGCTTCCATATCGCTTATAT TTTAATATCGTCTATATAGATTCCTGGACG

 TCTTTTAAAGCAGTTGATATCAAGACATCT AACCTCATTATTAAATTTCACTACTACTGC

Nucleotide sequence according to the graphical representation for each DRT:


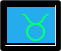
 GTTTTAGAGCTGTGCGGTTATTATGCTAGGGCACCG


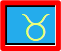
 GTTTTAGAGCTGTGCTGTTATTATGCTAGGACATCA


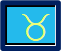
 GTTTTAAAGCTGTGCTGTTATTATGCTAGGGCACCA


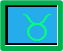
 GTTTTAGAGCTGCGCGGTTATTATGCTATGCTAGGA


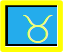
 GTTTTAGAGCTGTGCTGTTATTATGCTAGGGCACCA
