# Supplementary material for: Group B Streptococcus CRISPR1 Typing of Maternal, Fetal, and Neonatal Infectious Disease Isolates Highlights the Importance of CC1 in In Utero Fetal Death
Source: Microbiol Spectr. 2023 Jun 21;11(4):e05221-22. doi: 10.1128/spectrum.05221-22 (PMC10434043; doi:10.1128/spectrum.05221-22)
Supplement: Supplemental file 2 — Table S2. Download spectrum.05221-22-s0002.pdf, PDF file, 0.5 MB [file spectrum.05221-22-s0002.pdf]

Table S2 : The table regroups all data for each isolate, including isolate number, date of isolation, source, origin, graphic representation of CRISPR typing result, CC interpretation of CRISPR1 typing, SNP typing result and CC interpretation of SNP typing.

**Isolate number**	**Date of isolation**	**Adult / Neonate[a]**	**Source[b]**	**Spacers**	**DRT**	**CC (CRISPR)**	**SNP profile[c]**	**CC (SNP)**
H04-198836	00/00/2004	N	Blood			CC1	CTAG	CC1
H04-181936	00/00/2004	N	Blood			CC1	CTAG	CC1
H05-194154	13/09/2005	N	Blood			CC1	CTAG	CC1
LA07-112323	04/01/2007	N	Joint fluid			CC1	CTAG	CC1
P09-156898	00/00/2009	N	Placenta			CC1	CTAG	CC1
P10-152346	00/00/2010	N	Placenta			CC1	CTAG	CC1
190007	12/01/2011	N	Blood			CC1	CTAG	CC1
180111	26/06/2012	A	Blood			CC1	CTAG	CC1
157609	26/06/2012	A	Placenta			CC1	CTAG	CC1
181426	17/07/2012	N	Blood			CC1	CTAG	CC1
196208	05/03/2014	N	Blood			CC1	CTAG	CC1
158462	05/03/2014	N	Placenta			CC1	CTAG	CC1
153387	03/08/2014	N	Placenta			CC1	CTAG	CC1
185026	24/06/2015	N	Blood			CC1	CTAG	CC1
106518	01/07/2016	N	Placenta			CC1	CTAG	CC1
192742	13/09/2017	A	Blood			CC1	CTAG	CC1
154617	20/09/2017	A	Breast abscess			CC1	CTAG	CC1
158099	07/08/2018	A	Breast abscess			CC1	CTAG	CC1
159968	18/09/2018	N	Placenta			CC1/19	CTAG	CC1
H04-191349	00/00/2004	N	Blood			CC10	CTAA	CC10
H04-181311	00/00/2004	N	Blood			CC10	CTAA	CC10
P10-159070	00/00/2010	N	Placenta			CC10	CTAA	CC10
155489	23/08/2015	A	Breast abscess			CC10	CTAA	CC10
191221	13/10/2015	N	Blood			CC10	CTAA	CC10
153089	03/12/2016	A	Placenta			CC10	CTAA	CC10
105497	22/09/2018	A	Breast abscess			CC10	CTAA	CC10
188362	28/03/2019	N	Blood			CC10	CTAA	CC10
196130	29/07/2019	N	Blood			CC10	CTAA	CC10
189110	21/06/2020	A	Blood			CC10	CTAA	CC10
**A909[d]**		N	Blood			CC10		
H04-187401	00/00/2004	N	Blood			CC17	CCGG	CC17
H04-189040	00/00/2004	N	Blood			CC17	CCGG	CC17
LCR04-128813	00/00/2004	N	CSF			CC17	CCGG	CC17
H04-197814	00/00/2004	N	Blood			CC17	CCGG	CC17
H04-181802	00/00/2004	N	Blood			CC17	CCGG	CC17
H04-182147	00/00/2004	N	Blood			CC17	CCGG	CC17
H05-182877	00/00/2005	N	Blood			CC17	CCGG	CC17
H05-181571	00/00/2005	N	Blood			CC17	CCGG	CC17
H05-196270	26/10/2005	N	Blood			CC17	CCGG	CC17
LCR05-119775	27/12/2005	N	CSF			CC17	CCGG	CC17
H05-199356	28/12/2005	N	Blood			CC17	CCGG	CC17
H06-191699	12/09/2006	N	Blood			CC17	CCGG	CC17
H07-187462	28/07/2007	N	Blood			CC17	CCGG	CC17
LCR07-119089	29/07/2007	N	CSF			CC17	CCGG	CC17
H07-188982	28/06/2007	N	Blood			CC17	CCGG	CC17
H07-191444	16/10/2007	N	Blood			CC17	CCGG	CC17
LCR08-113982	22/01/2008	N	CSF			CC17	CCGG	CC17
H08-196088	22/01/2008	N	Blood			CC17	CCGG	CC17
H09-187845	30/10/2009	N	Blood			CC17	CCGG	CC17
H10-198150	20/05/2010	N	Blood			CC17	CCGG	CC17
LA10-118094	30/06/2010	N	Joint fluid			CC17	CCGG	CC17
193361	15/03/2011	N	Blood			CC17	CCGG	CC17
114320	15/03/2011	N	CSF			CC17	CCGG	CC17
182429	04/08/2012	N	Blood			CC17	CCGG	CC17
186090	13/10/2012	A	Blood			CC17	CCGG	CC17

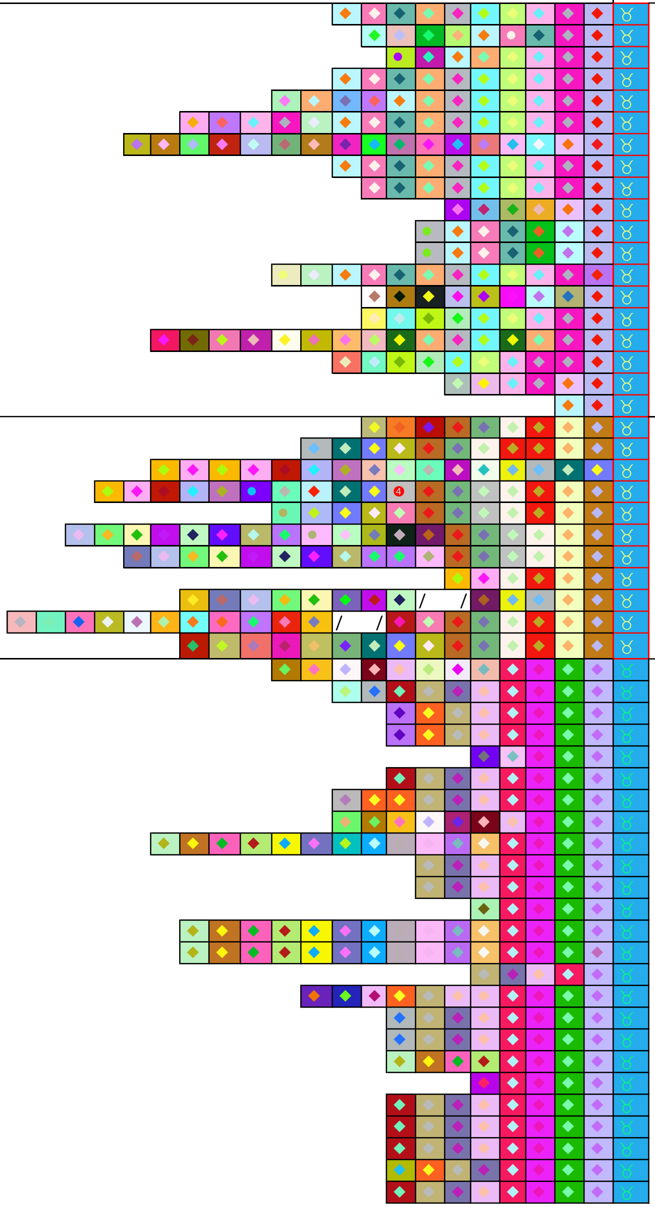

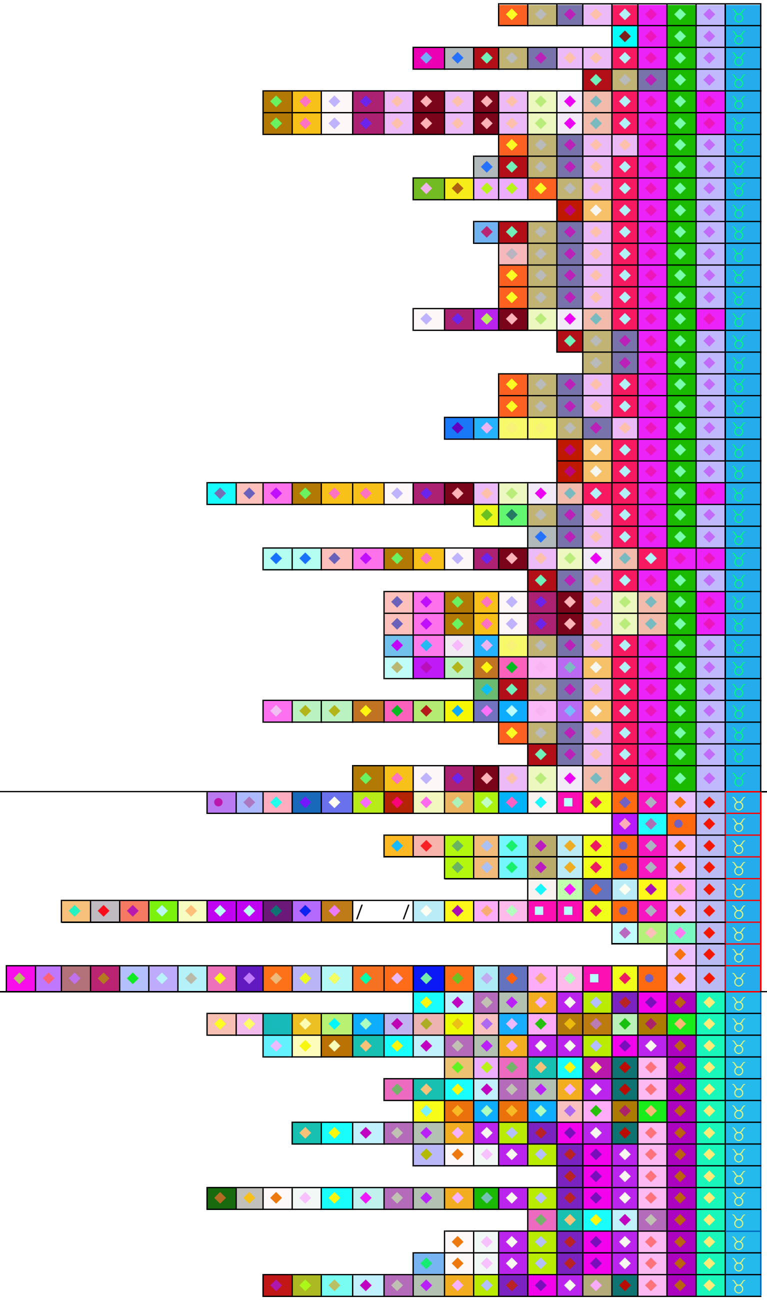

514602	12/11/2012	N	CSF	CC17	CCGG	CC17
190978	05/01/2013	N	Blood	CC17	CCGG	CC17
184044	14/08/2013	N	Blood	CC17	CCGG	CC17
154704	15/11/2013	A	Placenta	CC17	CCGG	CC17
189832	25/11/2013	N	Blood	CC17	CCGG	CC17
118812	25/11/2013	N	CSF	CC17	CCGG	CC17
193430	23/01/2014	N	Blood	CC17	CCGG	CC17
509807	28/02/2014	N	CSF	CC17	CCGG	CC17
195911	01/03/2014	N	Blood	CC17	CCGG	CC17
158522	07/03/2014	A	Breast abscess	CC17	CCGG	CC17
181796	05/06/2014	N	Blood	CC17	CCGG	CC17
183455	01/07/2014	N	Blood	CC17	CCGG	CC17
186770	25/07/2015	N	Blood	CC17	CCGG	CC17
192750	09/11/2015	N	Blood	CC17	CCGG	CC17
195509	24/12/2015	N	Blood	CC17	CCGG	CC17
510300	06/03/2016	N	CSF	CC17	CCGG	CC17
180113	07/03/2016	N	Blood	CC17	CCGG	CC17
184160	07/05/2016	N	Blood	CC17	CCGG	CC17
510783	07/05/2016	N	CSF	CC17	CCGG	CC17
157779	21/07/2016	A	Breast abscess	CC17	CCGG	CC17
197927	01/01/2017	A	Blood	CC17	CCGG	CC17
154005	01/01/2017	A	Placenta	CC17	CCGG	CC17
154724	20/01/2017	A	Breast abscess	CC17	CCGG	CC17
188969	15/05/2018	N	Blood	CC17	CCGG	CC17
194491	17/08/2018	N	Blood	CC17	CCGG	CC17
194726	22/08/2018	N	Blood	CC17	CCGG	CC17
195915	11/09/2018	N	Blood	CC17	CCGG	CC17
198553	25/10/2018	N	Blood	CC17	CCGG	CC17
115648	25/10/2018	N	Joint fluid	CC17	CCGG	CC17
164128	25/12/2018	N	Placenta	CC17	CCGG	CC17
184224	24/01/2019	N	Blood	CC17	CCGG	CC17
181202	29/02/2020	N	Blood	CC17	CCGG	CC17
182231	15/03/2020	N	Blood	CC17	CCGG	CC17
180171	09/07/2020	N	Blood	CC17	CCGG	CC17
187406	29/10/2020	N	Blood	CC17	CCGG	CC17
**BM110**[e]		N	Invasive infection	CC17		
H06-186617	00/00/2006	N	Blood	CC19	CCAG	CC19
182269	12/08/2010	A	Blood	CC19	CCAG	CC19
183458	04/08/2013	N	Blood	CC19	CCAG	CC19
505297	04/08/2013	N	CSF	CC19	CCAG	CC19
187545	08/08/2015	N	Blood	CC19	CCAG	CC19
184640	15/05/2016	N	Blood	CC19	CCAG	CC19
152593	02/04/2018	N	Placenta	CC1/19	CCAG	CC19
181661	02/08/2020	A	Blood	CC1/19	CCAG	CC19
**2603V/R**[f]				CC19		
H07-188640	00/00/2007	N	Blood	CC23	TCGG	CC23 and CC22
H08-191588	00/00/2008	N	Blood	CC23	TCGG	CC23 and CC22
H10-197635	00/00/2010	N	Blood	CC23	TCGG	CC23 and CC22
192614	02/03/2011	N	Blood	CC23	TCGG	CC23 and CC22
180152	28/06/2011	A	Blood	CC23	TCGG	CC23 and CC22
193271	07/02/2013	N	Blood	CC23	TCGG	CC23 and CC22
198998	20/04/2014	A	Blood	CC23	TCGG	CC23 and CC22
152364	06/07/2014	N	Placenta	CC23	TCGG	CC23 and CC22
183770	07/07/2014	N	Blood	CC23	TCGG	CC23 and CC22
188428	29/09/2014	N	Blood	CC23	TCGG	CC23 and CC22
180397	06/04/2015	N	Blood	CC23	TCGG	CC23 and CC22
187500	11/07/2016	N	Blood	CC23	TCGG	CC23 and CC22
199667	27/12/2017	N	Blood	CC23	TCGG	CC23 and CC22
189057	21/06/2020	A	Blood	CC23	TCGG	CC23 and CC22

**NEM316**[g]		N	Blood	CC23		
159418	14/02/2015	A	Breast abscess	CC22	TCGG	CC23 and CC22
152387	07/03/2016	N	Placenta	CC388	CCAG	CC19
187952	24/04/2018	A	Blood	CC388	CCGG	CC17
112043	10/09/2020	N	CSF	CC388	CCAG	CC19

[a]Adult = A / Neonate = N

bSource was Blood or Cerebrospinal fluid (CSF) or Placenta or Joint fluid or Breast abscess

[c]SNP are presented in this order : glnA36; glnA429; glcK180; adhP111

[d]Tettelin *et al* . 2005

[e]Musser *et al.* 1989

[f]Tettelin *et al.* 2002

[g]Glaser *et al.* 2002
